# Homozygosity disequilibrium associated with treatment response and its methylation regulation

**DOI:** 10.1186/s12919-018-0150-9

**Published:** 2018-09-17

**Authors:** Hsin-Chou Yang, Chia-Wei Chen

**Affiliations:** Institute of Statistical Science, Academia Sinica, No 128, Sec 2, Academia Rd, Nankang 115, Taipei, Taiwan

## Abstract

Homozygosity disequilibrium (HD), indicating a nonrandom pattern of sizable runs of homozygosity that deviates from a random allocation of homozygous and heterozygous genotypes in the genome, is an important phenomenon in population genomics and medical genomics. We performed the first genome-wide study investigating the roles of HD in pharmacogenomics and pharmacoepigenomics by analyzing GAW20 data. We inferred whole-genome profiles of homozygosity intensities and performed genome-wide homozygosity association analyses to identify regions of HD associated with triglyceride (TG) response to fenofibrate by using LOHAS (Loss-of-Heterozygosity Analysis Suite) software. The analysis identified a region of HD contained in *MACROD2* at 20p12 to be significantly associated with TG response to fenofibrate. We also examined the common genetic component in TG and methylation responses to fenofibrate. The methylation response to fenofibrate was regarded as a methylation quantitative trait, and our methylation quantitative trait locus analysis identified a *cis*-acting regulation association with marginal significance between the homozygosity intensity of *MACROD2* and the methylation response to fenofibrate. These findings may help delineate the genetic basis of pharmacogenomic and pharmacoepigenomic responses to fenofibrate intervention.

## Background

Homozygosity disequilibrium (HD), coined by Yang et al. [1], indicates a nonrandom pattern of sizable runs of homozygosity that deviates from a random allocation of homozygous and heterozygous genotypes in the genome. The major genetic mechanisms of HD include, but are not limited to, autozygosity [2], natural selection [3], and chromosomal aberrations [4]. HD is natural variation among individuals and has interethnic differences [5, 6]. HD has familial aggregation, suggesting a genetic component of HD. Genomic distribution of HD in humans has been characterized [5, 7, 8]. Genetic contributions of HD to the susceptibility of Mendelian diseases, complex disorders, and cancers have been reviewed [9]; HD is especially crucial for neurodevelopment-related diseases [10, 11] and autoimmune diseases [1, 12]. Gene regulation of disease-associated HD also has been observed [8].

Previously, we developed statistical method and software (Loss-of-Heterozygosity Analysis Suite [LOHAS]) to detect HD based on genotypes of single nucleotide polymorphisms (SNPs) in SNP microarrays [5]. LOHAS was applied to investigate genetic association between HD and disease susceptibility [1, 5, 13] and the relationship between HD and continental populations [5]. We also developed another method and software (AF/LOH/LCSH/AI/CNV/CNA Enterprise [ALICE]) to detect HD through a whole-genome SNP hybridization intensity analysis [14].

Because no studies had investigated HD by using whole-genome sequencing data, LOHAS was extended to analyze the whole-genome sequencing data set in GAW18 [7]. The extension of LOHAS was based on the assumption that all rare variants (RVs) have an equal weight, even though common homozygotes of RVs with a lower minor allele frequency carry less homozygosity information [7]. In GAW19, we further enhanced LOHAS by considering a local property and genetic information of homozygosity in the homozygosity intensity estimation. In contrast to the previous estimation procedure, the new method did not assume equal importance of RVs when defining HD. In addition to the higher computational efficiency, simulation studies suggested the new method has well-controlled type 1 error and higher power than our previous homozygosity association test [7]. The new method not only identified the regions of HD associated with blood pressure, but also discovered unreported evidence of gene regulation by the regions of HD associated with blood pressure.

No studies had investigated the role of HD in pharmacogenomics and pharmacoepigenomics. GAW20 provides a real data set from the Genetics of Lipid Lowering Drugs and Diet Network (GOLDN) project [15]. The data set consists of treatment response, and whole-genome genotypes of SNPs and whole-genome methylation levels of cytosine-phosphate-guanine (CpG) sites, providing an unmet opportunity to study HD in pharmacogenomics and pharmacoepigenomics. This study aims to evaluate the effects of HD on the treatment responses to fenofibrate (i.e., triglyceride [TG] and DNA methylation changes resulting from fenofibrate intervention) and methylation regulation.

## Methods

### Materials

GAW20 provides clinical variables [TG (mg/dL) measurements before and after fenofibrate intervention] and covariates [sex, age, field center, smoking status, and two metabolic syndrome indices—Adult Treatment Panel (ATP) and International Diabetes Federation (IDF)] for 1105 individuals, derived from 188 independent pedigrees in the GOLDN project. Among the 1105 individuals, 1105 and 818 individuals had TG measurements before and after fenofibrate intervention, respectively. GAW20 provides a whole-genome genotype data set of 718,542 autosomal SNPs for 822 individuals, derived from 173 independent pedigrees. The SNP data were obtained using the Affymetrix 6.0 array. SNPs were removed if they violated Mendelian segregation, had a minor allele frequency of < 0.01 or a call rate of < 96%, or failed the Hardy-Weinberg equilibrium test in *p* < 1 × 10^− 6^. Details of quality control can refer to the GOLDN project [15]. GAW20 also provides whole-genome methylation pre- and post-fenofibrate treatment constituting of 463,995 CpG sites for 446 individuals from 140 independent pedigrees. The methylation data were obtained using the Illumina Infinium Human Methylation450 BeadChip. CpG sites were removed if they had insignificant detection *p* value, mismatch with annotation file, or were close to SNPs. Samples were removed if they had missing data of > 1.5% of CpG sites and were outliers in principal component of T-cell purity. Combat normalization was used to correct for batch effect. For details of quality control, refer to the GOLDN project [15].

### Statistical methods

We applied the double-weight local polynomial model [8] in LOHAS to estimate homozygosity intensities of 822 individuals. Sliding windows on each chromosome were constructed by using the nearest-neighbor method. The number of SNPs in each sliding window was 5% of SNPs on a chromosome. A cubic kernel weight and a locus weight with a threshold of minor allele frequency of 0.05 were considered. The estimates of homozygosity intensity range from 0 to 1. A higher value indicates a higher homozygosity.

In each sliding window, generalized estimation equation (GEE) analysis was performed to examine the relationship between homozygosity intensities and TG responses to fenofibrate, with concomitant adjustment for covariates (sex, age, field center, smoking status, and two metabolic syndrome indices—ATP and IDF) of 778 individuals. To consider potential population stratification, we performed principle component analysis based on a linkage disequilibrium pruned set of 80,930 SNPs at *r*^2^ < 0.2. The top 10 principal components were included as covariates in the GEE analysis. In this study, TG response to fenofibrate was measured by a reduction of TG after fenofibrate intervention (i.e., the average of two TG measurements after fenofibrate intervention [visit 3 and visit 4] minus the average of two TG measurements before fenofibrate intervention [visit 1 and visit 2]). In general, fenofibrate intervention has a TG-lowering effect.

Bonferroni correction was performed for a multiple-testing problem in this GEE-based genome-wide homozygosity association study. Because of a high dependency of statistical tests across overlapping sliding windows, we estimated the effective number of independent tests (*n*_*e*_) by adapting the method in Li et al. [16] and then adjusted for multiple tests as follows: Let *n*_*j*_ denote the number of the homozygosity association tests (i.e., the number of sliding windows) on autosome *j*. Let *I*[*A*] denote an indicator taking a value of 1 if event *A* holds and 0 otherwise. By each autosome, we estimated the effective number of independent tests *n*_*e*, *j*_ using ${n}_j-{\sum}_{i=1,\cdots, {n}_j}\left\{I\left[{\lambda}_{i,j}>1\right]\left({\lambda}_{i,j}-1\right)\right\}$, where {*λ*_*i*, *j*_, *i* = 1, ⋯, *n*_*j*_} indicates the eigenvalues of the correlation matrix of homozygosity intensities of sliding windows on autosome *j*. Finally, we used *α*/∑_*j*_*n*_*e*, *j*_ as the critical significance level with *α* = 0.05.

The changes in TG and DNA methylation patterns resulting from fenofibrate intervention were found, but no association was observed between the TG and methylation responses in previous studies [17]. We examined whether the treatment responses in TG and DNA methylation were associated with the common genetic component. Once the regions of HD associated with the TG response to fenofibrate were identified, we further examined whether the identified regions of HD were also associated with the methylation response to fenofibrate. Similar to the homozygosity association study for the TG response to fenofibrate, GEE analysis was performed to examine the relationship between homozygosity intensities and methylation response to fenofibrate with concomitant adjustment for covariates (sex, age, field center, smoking status, the top 10 principal components, and two metabolic syndrome indices—ATP and IDF) of 429 individuals. The *cis*-acting and *trans*-acting methylation quantitative trait locus (meQTL) analyses were performed, where the methylation quantitative trait was the methylation response to fenofibrate. Before the meQTL analyses, a further methylation normalization was performed. Because a probe-type bias of Infinium I versus Infinium II was observed in the normalized data of GAW20, we performed the beta-mixture quantile normalization method [18] based on the methylation beta values (i.e., the ratio of methylated to combined intensity values) and then transformed the beta values to M values (i.e., log_2_ ratio of beta value to 1 − beta value). The methylation response to fenofibrate was calculated by a change of M values after fenofibrate intervention (i.e., M values at visit 4 minus M values at visit 2).

## Results

We estimated the whole-genome profiles of homozygosity intensities of 822 individuals. We obtained all regions of HD satisfying homozygosity intensity of ≥0.9 and region length of ≥5 Mb in the genomes of all individuals. The minimum, first quartile, second quartile, third quartile, and maximum of lengths of regions of HD were 5.01 Mb, 8.82 Mb, 11.06 Mb, 12.90 Mb, and 40.91 Mb, respectively. We also calculated the total length of regions of HD in each genome. The minimum, first quartile, second quartile, third quartile, and maximum of total length of regions of HD carried by an individual were 5.15 Mb, 9.44 Mb, 12.46 Mb, 23.38 Mb, and 168.05 Mb, respectively.

**Fig. 1:**
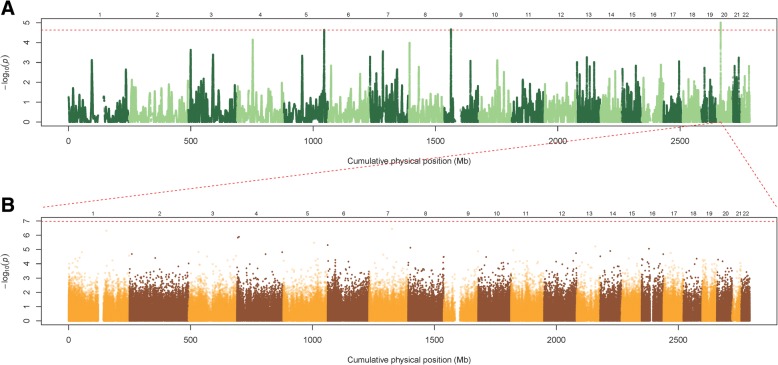
Genome-wide homozygosity association analysis and meQTL analysis, with concomitant adjustment for sex, age, field center, smoking status, ATP, and 10 principal components. **a**, The results of genome-wide homozygosity association tests for TG response to fenofibrate are shown in a Manhattan plot. The vertical axis represents the *p* values (−log_10_ scale) of the homozygosity association tests. The horizontal axis represents the physical positions of the anchor SNPs of sliding windows by chromosome. **b**, The results of meQTL analyses for the fenofibrate-associated HD on *MACROD2* in chromosome 20p12. The vertical axis represents the *p* values (−log_10_ scale) of the association tests. The horizontal axis represents the physical positions of the CpG sites by chromosome

We performed two genome-wide homozygosity association studies to identify fenofibrate response-associated regions of HD. In the first study, a GEE analysis with concomitant adjustment for covariates sex, age, field center, smoking status, ATP, and 10 principal components were performed based on 778 individuals. Figure 1a shows the Manhattan plot. For a multiple-testing correction, we estimated the effective number of independent tests was 2145. After a Bonferroni correction, this study identified three genomic regions strongly associated with a TG-lowering effect to fenofibrate intervention. The most significant sliding window at each of the three regions was anchored at rs254239 (chr5:164916163, *p* = 2.308 × 10^− 5^), rs7037978 (chr9:27330668, *p* = 2.15 × 10^− 5^), and rs17704829 (chr20:15691912, *p* = 9.86 × 10^− 6^). In addition to a number of significant principal components, field center (Utah vs. Minnesota) was the only significant covariate in one of the three significant sliding window; *p* = 0.0309 in the significant sliding window anchor at rs17704829.

**Fig. 2:**
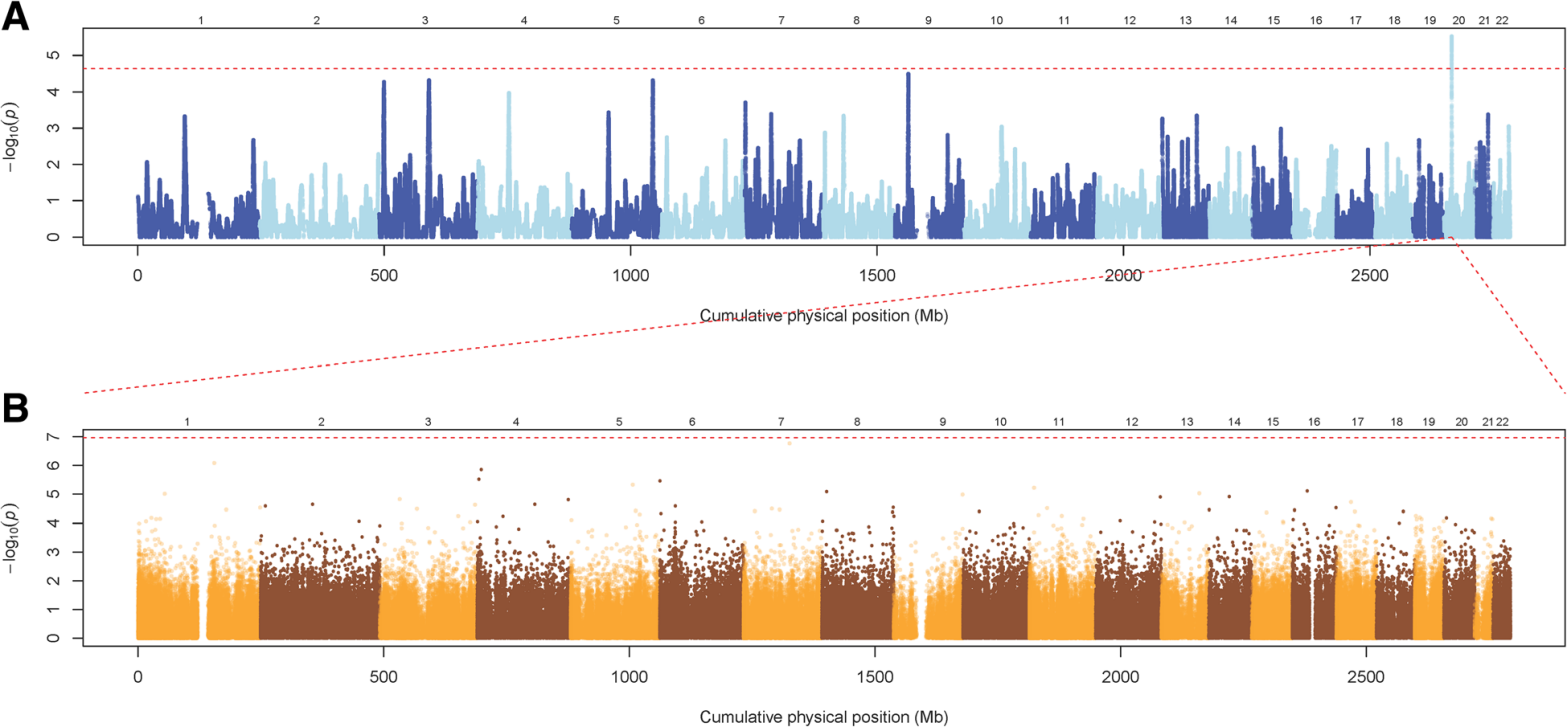
Genome-wide homozygosity association analysis and meQTL analysis, with concomitant adjustment for sex, age, field center, smoking status, IDF, and 10 principal components. **a**, The results of genome-wide homozygosity association tests for TG response to fenofibrate are shown in a Manhattan plot. The vertical axis represents the *p* values (−log_10_ scale) of the homozygosity association tests. The horizontal axis represents the physical positions of the anchor SNPs of sliding windows by chromosome. **b**, The results of meQTL analyses for the fenofibrate-associated HD on *MACROD2* in chromosome 20p12. The vertical axis represents the *p* values (−log_10_ scale) of the association tests. The horizontal axis represents the physical positions of the CpG sites by chromosome

In the second study, the GEE model was similar to the previous model except for replacing ATP with IDF. The second study only identified one window as associated with a TG-lowering effect to fenofibrate intervention; the anchor SNP was rs764140 (chr20:15713167, *p* = 2.95 × 10^− 6^) (Fig. 2a). Both of the studies identified *MACROD2* (MACRO domain containing 2) in chromosome 20p12 as an important gene region associated with treatment response to fenofibrate. In addition to a number of significant principal components, field center (Utah vs Minnesota) was the only significant covariate; *p* = 0.0306 in the significant sliding window anchor at rs764140.

To investigate the common genetic component in the TG and methylation responses to fenofibrate, we performed a *cis*-meQTL analysis for the two fenofibrate response-associated windows of HD anchor at rs17704829 and rs764140 on *MACROD2*. In total, 24 CpG sites on *MACROD2* were provided in the Illumina Infinium Human Methylation450 BeadChip. Homozygosity intensities of the two windows were marginally significantly associated with the methylation response to fenofibrate at two CpG sites: cg07953890 (chr20:13976782; *p* = 2.318 × 10^− 2^ for the window anchor at rs17704829; *p* = 2.555 × 10^− 2^ for the window anchor at rs764140) and cg09937190 (chr20:15177509; *p* = 3.614 × 10^− 2^ for the window anchor at rs17704829; *p* = 3.911 × 10^− 2^ for the window anchor at rs764140). To identify more meQTL associations, we also performed *trans*-meQTL analyses. The results are summarized in Fig. 1b and Fig. 2b. However, no *trans*-acting regulation association was identified after a multiple-testing correction.

## Discussion and conclusions

We performed the first genome-wide study investigating the role of HD in pharmacogenomics and pharmacoepigenomics. The homozygosity intensity estimation method and homozygosity association tests in LOHAS software [5] (http://www.stat.sinica.edu.tw/hsinchou/genetics/loh/LOHAS.htm) we used are proven useful tools for studying HD in simulation studies and real data analyses [1, 5, 7, 8, 13]. In this study, we inferred whole-genome profiles of homozygosity intensities and examined the genomic distributions of HD. Our two genome-wide homozygosity association analyses pinpointed the same region of HD, contained in *MACROD2* at 20p12, strongly associated with TG response to fenofibrate. *MACROD2* was reported to be associated with metabolic diseases such as hypertension [19], as well as drug resistance such as tamoxifen for breast cancers [20]. Our meQTL analysis identified a *cis*-acting regulation association with marginal significance between the homozygosity intensity of *MACROD2* and the methylation response to fenofibrate. These findings may help delineate the genetic basis of pharmacogenomic and pharmacoepigenomic responses to fenofibrate.
